# Bovine and Human Serum Albumin Interactions with 3-Carboxyphenoxathiin Studied by Fluorescence and Circular Dichroism Spectroscopy

**DOI:** 10.3390/molecules15063905

**Published:** 2010-06-01

**Authors:** Aurica Varlan, Mihaela Hillebrand

**Affiliations:** Department of Physical Chemistry, University of Bucharest, Bd. Regina Elisabeta, 4-12, Bucharest, Romania; E-Mail: mihh@gw-chimie.math.unibuc.ro

**Keywords:** steady state fluorescence, synchronous fluorescence, circular dichroism, 3-carboxyphenoxathiin, serum albumin

## Abstract

The interactions of 3-carboxyphenoxathiin with Bovine Serum Albumin (BSA) and Human Serum Albumin (HSA) have been studied by fluorescence and circular dichroism spectroscopy. The binding of 3-carboxyphenoxathiin quenches the BSA and HSA fluorescence, revealing a 1:1 interaction with a binding constant of about 10^5^ M^-1^. In addition, according to the synchronous fluorescence spectra of BSA and HSA in presence of 3-carboxyphenoxathiin, the tryptophan residues of the proteins are most perturbed by the binding process. Finally, the distance between the acceptor, 3-carboxyphenoxathiin, and the donor, BSA or HSA, was estimated on the basis of the Förster resonance energy transfer (FRET). The fluorescence results are correlated with those obtained from the circular dichroism spectra, which reveal the change of the albumin conformation during the interaction process.

## 1. Introduction

Serum albumins, especially bovine (BSA) and human (HSA), labeled with fluorescent probes are commonly used for the investigation of surface induced conformational changes in protein interfaces. From a biopharmaceutical point of view, one of the most important biological functions of albumins is their ability to carry drugs, endogenous and exogenous substances. Numerous experiments with the aim of characterizing the binding capacity and binding sites of albumins have been carried out. The spectral changes observed on the binding of fluorophores with proteins are important tools for the investigations of the topology of the binding sites, of the conformational changes and for the characterization of substrate to ligand binding [1]. The determination of protein quantity in biological liquids is of great importance in biology and medicine [2] and fluorescent probes are successfully applied for this approach [3]. Serum albumin being the major transporters binding protein for the drugs and other physiological substances, it is considered as a model for studying drug–protein interaction *in vitro*. On the other hand, among the fluorophores, the aromatic or heteroaromatic carboxylic acids have been found suitable for this purpose [4].

The interactions of carboxylic acids with albumins are interesting from several points of view. Firstly, at physiological pH, they are usually in the form of carboxylate ion and, therefore, can provide information on the electrostatic interactions. Secondly, they present enhanced water solubility at physiological pH.

**Figure 1:**
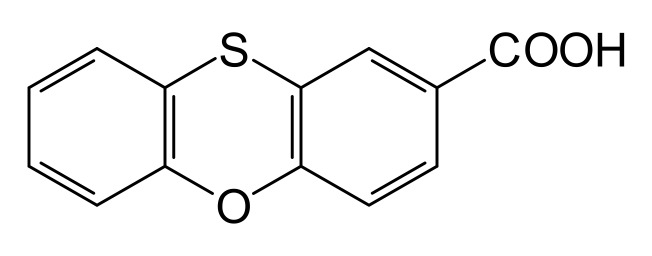
The structure of 3-carboxyphenoxathiin (**I**).

In this paper we report the experimental study using steady state fluorescence and circular dichroism spectroscopy of the interaction of BSA/HSA with a carboxylic acid, 3-carboxyphenoxathiin (**I,** Figure 1). The pK_a_ of the acid-base equilibrium of **I** is 4.57. Therefore, at pH 7.4 we assume that the predominant form is the dissociated one.

We have focused on this compound for two reasons. Firstly, we were mostly interested in using as a model for protein-binding processes a compound for which the excited state properties are already known, as well as its behavior in the change of the local polarity as reflected in its interaction with cyclodextrins [5,6]. Secondly, its fluorescence emission is in a wavelength domain well separated from the protein one. We already have some experience in the field of carboxylic acids, as two studies concerning a related compound, 2-carboxyphenoxathiin, were already published [7,8]. The influence of **I** on the intrinsic albumin fluorescence was investigated at 345 nm for HSA, and 346 nm for BSA. When the ligand is sufficiently close to the tryptophan and tyrosine fluorescent residues, a fluorescence quenching can be observed. The binding constants and the number of binding sites were derived. In addition, the conformational changes of BSA and HSA upon interaction are discussed on the basis of synchronous fluorescence spectra and circular dichroism.

## 2. Results and Discussion

### 2.1. Fluorescence Spectra of the **I**–Albumin Systems

**Figure 2:**
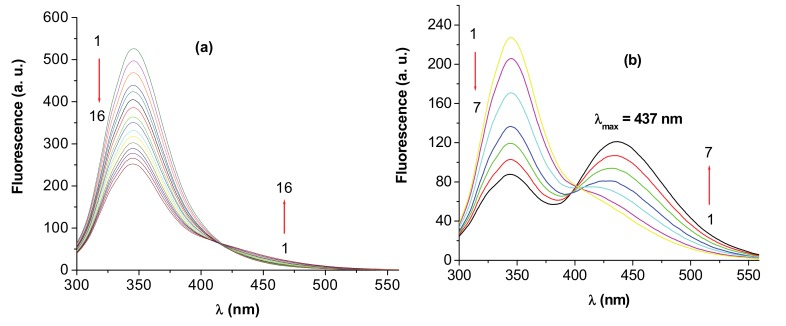
Emission spectra of BSA in the presence of different concentrations of **I**; [BSA] = 3 × 10^-6^ M; (a) 1–16: d/p = 0–1; (b) 1–7: d/p = 1–5; λ_ex_ = 286 nm; pH = 7.4.

Fluorescence quenching refers to any process, which decreases the fluorescence intensity of a sample. A variety of molecular interactions can result in quenching, including excited-state reactions, molecular rearrangements, energy transfer, ground-state complex formation and collisional quenching [9]. When the concentrations of BSA/HSA were fixed at 3×10^-6^ M, and the concentration of **I** was gradually increased, the fluorescence intensity of the protein bands, BSA and HSA, decreased. As an example, the family of curves obtained for BSA and **I** is presented in Figure 2.

In this case, up to a ligand (drug)/protein molar ratio (d/p) of about 1.0 the main effect was the quenching of the protein fluorescence, as evidenced by the spectra in Figure 2a. At larger ligand concentration, Figure 2b, a new fluorescence emission can be observed, initially as a shoulder and afterwards as a band continuously red shifted towards 437 nm, the maximum position of the free ligand emission. A shift of the emission band of **I** was previously observed in the presence of cyclodextrin [6] but in an opposite sense, *i.e.* from 437 nm, the emission maximum of the free ligand, towards 419 nm, the maximum of the included ligand in the cyclodextrin cavity at full complexation. The hydrophobic environment around the included species provided by the cyclodextrin cavity explained this hypsochromic shift. The comparison of the present experimental data with these previous observations in the presence of cyclodextrin leads to the following conclusions. At low ligand concentration, the albumin-complexed ligand is likely the predominant species in solution; the maximum of the ligand band, similar with that in the cyclodextrin complexes, reflects the position of the ligand inside a more hydrophobic region of the albumin. Increasing the ligand concentration, the uncomplexed form becomes predominant and the maximum emission wavelength reaches the values for the free ion. The presence of the fluorescence maximum of the free carboxylate ion shows that no modification (protonation) of the ligand occurs during the process.

### 2.2. Stern-Volmer Analysis

In most cases, the albumin fluorescence quenching in the presence of several ligands is characterized by a linear Stern-Volmer (SV) plot and is usually analyzed using the classical Stern-Volmer (SV) Equation (1) [7]:

(1) $$\frac{F_{0}}{F}=1+K_{SV}[Q]$$

where F_0_ and F are the steady state fluorescence intensities at the maximum wavelength in the absence and presence of quencher, respectively, [Q] is the quencher concentration and K_SV_ is the Stern-Volmer constant. However, in our case, for both proteins, **I** determines an upward curvature of the plots, suggesting a more complex quenching process.

**Figure 3:**
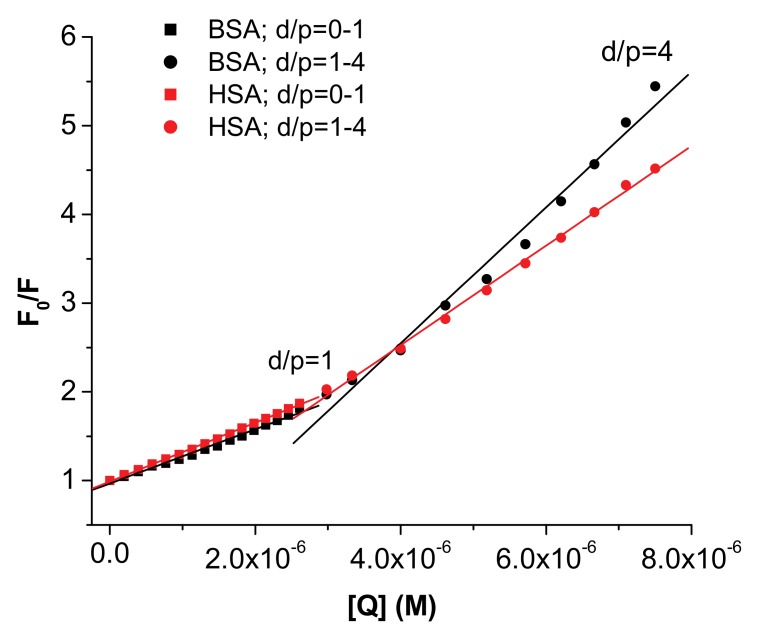
Two linear segments in Stern-Volmer plots for the quenching of HSA/BSA with **I**; [BSA]=[HSA] = 3×10^-6^M; λ_ex_ = 286 nm; λ_em_ = 345 nm; first segment d/p<1 (r^2^ = 0.999); second segment d/p>1 (r^2^ = 0.997).

**Figure 4:**
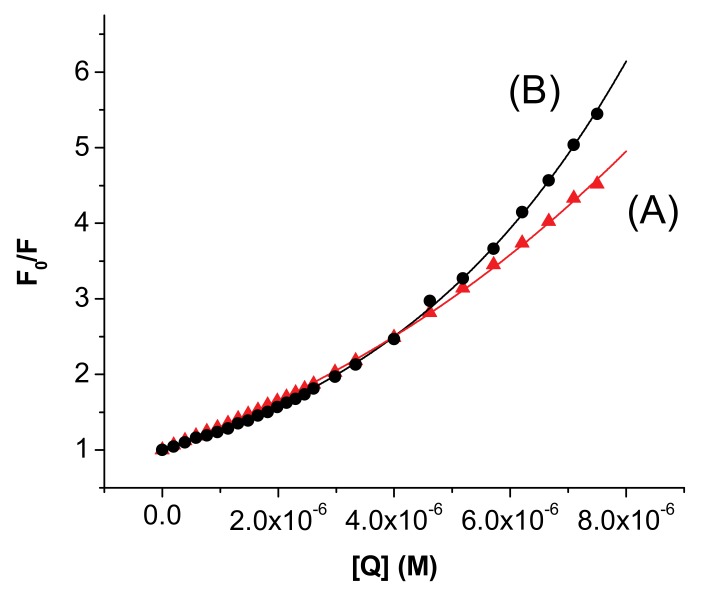
Stern-Volmer plots for the systems HSA—**I** (A) and BSA–**I** (B); [HSA] = [BSA] = 3×10^-6^ (M); A) r^2^ = 0.999; B) r^2^ = 0.999; the plots represent the best fits of the experimental points using Equation (2).

An upward curvature of the Stern-Volmer plots has also been previously reported for other ligand–albumin systems [10,11,12,13,14,15,16,17,18,19,20,21,22,23,24,25] and analyzed in different ways. One-way is to rationalize the data considering only the first linear domain of the Stern-Volmer plot, Equation (1), [17,18,19,20,21,22,23], or two linear segments [24,25]. Another method is to use the modified form of the Stern-Volmer equation when both dynamic and static quenching are operative, considering either the exponential form, the model of the sphere of action, Equation (2) [11,12,13], or the quadratic dependence [14,15,16] of the quencher concentration:

(2) $$F_{0}/F=\{1+K_{SV}*[Q]\}*\exp(V*[Q])$$

where *F_0_*, *F*, [Q] and K_SV_ have the previously defined meaning and V is the volume of the sphere of action. In our case, we followed both procedures and the fitted plots are presented in Figure 3 and Figure 4, the constants being included in Table 1.

It can be remarked that the change of the slope occurs at a drug/protein ratio, d/p ≈ 1, the SV constants being slightly increased at larger d/p values. Another observation refers to the values of V that lead to rather large values for the radius of the sphere of action. The statistical criteria are very good for both fitting procedures; it is therefore difficult to ascertain the type of the quenching, but in any case there is a static component.

Considering the Stern-Volmer constant of about 10^5^ M^-1^ and the lifetime of albumins of 10^-8^ s [16] we obtain a value of 10^13^ M^-1^ s^-1^ for the rate constant of the bimolecular quenching process, which largely overrides the accepted limit of the rate constant of the diffusional quenching implying biopolymers, 2×10^10^ M^-1^ s^-1^. This observation supports the fact that the experimental quenching of albumin fluorescence is due to a predominantly static process. According to literature data [17] we can suppose that the negative charge of **I** enhances the quenching process.

### 2.3. Determination of the Binding Parameters

Several models are given in literature for the determination of the binding parameters, the number of sites and the binding constants, considering one or several classes of binding [26,27,28,29,30,31,32,33]. Generally, all the models start from the Scatchard equation [26] for a single class of n independent binding sites, eq. (3):

(3) $$\nu =\frac{n\times K\times [L_{f}]}{\left(1+K\times [L_{f}]\right)}$$

where *ν* represents the binding ratio, *i.e.* the ratio of the bound ligand, [L_b_], to the total protein [P_t_], [L_f_] is the concentration of the free ligand at equilibrium, K is the association constant and n the number of binding sites. Working on the protein band, the calculation of *ν* is made considering that the measured fluorescence, F, at a given ligand concentration, is due to the unbound protein, [P]:

(4) $$\frac{F_{0}}{F}=\frac{\left[P_{t}\right]}{\left[P\right]}$$

(5) $$\nu =\frac{\left[L_{b}\right]}{\left[P_{t}\right]}=\frac{\left([L_{t}]-[L_{f}]\right)}{\left[P_{t}\right]}=n\times \frac{\left([P_{t}]-[P]\right)}{\left[P_{t}\right]}=n\times \frac{\left(F_{0}-F\right)}{F_{0}}$$

where [L_t_], [P_t_] are the total (analytical) concentrations of the ligand and the albumin, respectively. The main problem of the Scatchard equation is that in some models, the free ligand concentration, [L_f_] is replaced by the total concentration, *i.e.* by the amount of the added ligand, [L_f_] = [L_t_], approximation that is not always valid. In the following, in order to rationalize our experimental data on the albumin–carboxyphenoxathiin systems, we have focused on some of these equations, either in the linear or nonlinear forms, in which such approximations are avoided, Equations (6–7).

(6) $$log\frac{F_{0}-F}{F}=n\times logK-n\times log\frac{1}{\left[L_{t}]-\frac{F_{0}-F}{F_{0}}\times [P_{t}\right]}$$

(7) $$\frac{F_{0}-F}{F_{0}}=\frac{1}{2}\left(1+\frac{1}{K\times n\times [P_{t}]}+\frac{\left[L_{t}\right]}{n\times [P_{t}]}\right)-\frac{1}{2}\sqrt{{\left(1+\frac{1}{K\times n\times [P_{t}]}+\frac{\left[L_{t}\right]}{n\times [P_{t}]}\right)}^{2}-\frac{4\times [L_{t}]}{n\times [P_{t}]}}$$

The slope of the linear plot of log (F_0_-F)/F *vs.* log (1/([L_t_] –(F_0_-F)×[P_t_]/F_0_)), Equation (6), gives the number of sites and the intercept with the ordinate is the product n×log K. Using the linear regression analysis, Equation (6), for both proteins, two linear segments were obtained and, as in the case of the SV plots, the change of the slope occurs around d/p = 1.

**Figure 5:**
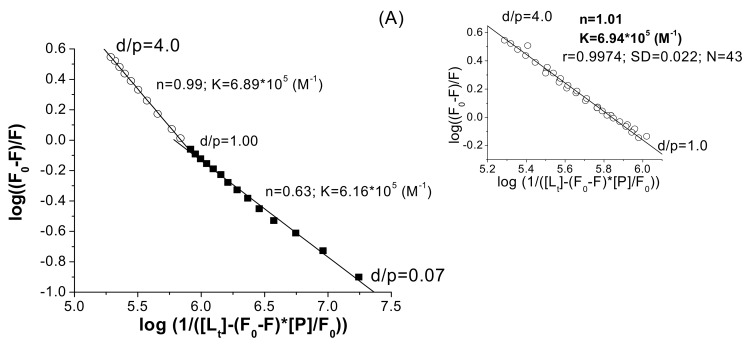
(A) Two linear segments fitting plot for the HSA–**I** system to Equation (6); [HSA] = 3×10^-6^ M; λ_ex_ = 286 nm; λ_em_ = 345 nm; inset, a triplicate experiments for d/p ≥ 1.0, fitted to Equation (6). (B) Two linear segments fitting plot for the BSA–**I** system to Equation (6); [BSA] = 3×10^-6^ M; λ_ex_ = 286 nm; λ_em_ = 346 nm.

**Figure 6:**
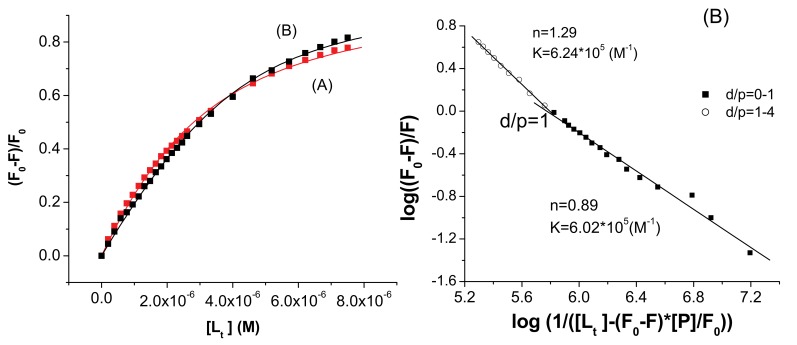
Nonlinear fitting of experimental data for the system (A) HSA–**I** (r^2^ = 0.998) and (B) BSA-**I** (r^2^ = 0.996) to Equation (7).

An example is given in Figure 5 for the HSA-**I** system. Above d/p = 1, the agreement between different experiments was very good as can be seen from the inset of Figure 4, in which the points obtained from a triplicate experiment are displayed. The averaged *n* and *K* values are included in Table 2. The *n* value close to 1 shows a one to one interaction, presumably near the Trp 214/212 residues. Using the nonlinear regression analysis (Figure 6), the separation of the two domains is not evident, but the value of *n* is lower than 1 for HSA, and slightly larger than 1 for the BSA–ligand system.

The values of the association constant obtained by the two methods of processing the experimental data are in good qualitative agreement especially for HSA and attest a strong interaction between the 3-phenoxathiin carboxylate ion and the bovine and human serum albumins.

### 2.4. Synchronous Fluorescence Spectroscopy

The synchronous fluorescence spectra can provide information about the molecular environment in the vicinity of the chromophore molecules. In the synchronous fluorescence spectra, the sensitivity associated with fluorescence is maintained while several advantages are available: spectral simplification, spectral bandwidth reduction and avoidance of different perturbing effects. 

**Figure 7:**
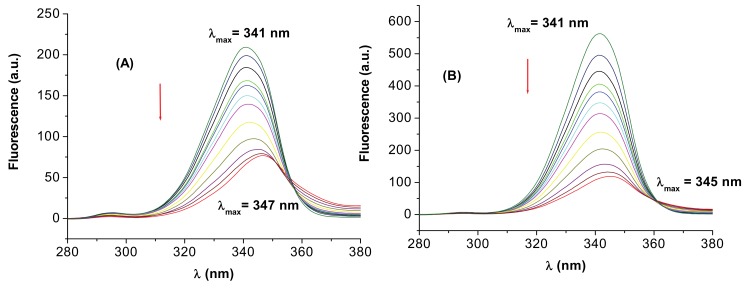
The effect of addition of **I** on the synchronous fluorescence spectrum of HSA (A) and BSA (B); Δλ = 60 nm.

The fluorescence spectrum of BSA/HSA mainly due to the Trp residues is sensitive to the microenvironment of these chromophores, the maximum emission wavelength being very useful in estimating the hydrophobicity around the tryptophan residues. The shift in the position of fluorescence emission maximum corresponds to changes of the polarity around the chromophore molecule. A blue shift of λ_max_ means that the amino acid residues are located in a more hydrophobic environment, and are less exposed to the solvent, while a red shift of λ_max_ implies that the amino acid residues are in a polar environment and are more exposed to the solvent [34,35,36,37,38]. The conformational changes of HSA caused by the 3-carboxyphenoxathiin binding were evaluated by measuring the synchronous fluorescence intensity of BSA/HSA before and during the addition of the compound. According to literature data [34], the synchronous fluorescence spectra were obtained considering the wavelength intervals Δλ = 15 nm and Δλ = 60 nm to evidence the tyrosine and tryptophan residues, respectively (Δλ = λ_em_ – λ_ex_). The synchronous fluorescence spectra for HSA and **I** is displayed in Figure 7 for Δλ = 60 nm.

The spectra evidence a shift from 341 to 347 nm showing that there are changes in conformation near tryptophan. The red shifts of the maximum emission wavelength of the tryptophan residue suggest that the interaction of HSA with **I** resulted in a more polar environment for tryptophan residue. The same effect is also observed for the system BSA and **I**. The polarity around tryptophan residues was increased and the hydrophobicity was decreased. Comparatively, using Δλ = 15 nm (data not shown) the maximum wavelength was practically unchanged reflecting little transformation around tyrosine.

### 2.5. Energy Transfer between 3-carboxyphenoxathiin and BSA/ HSA

**Figure 8:**
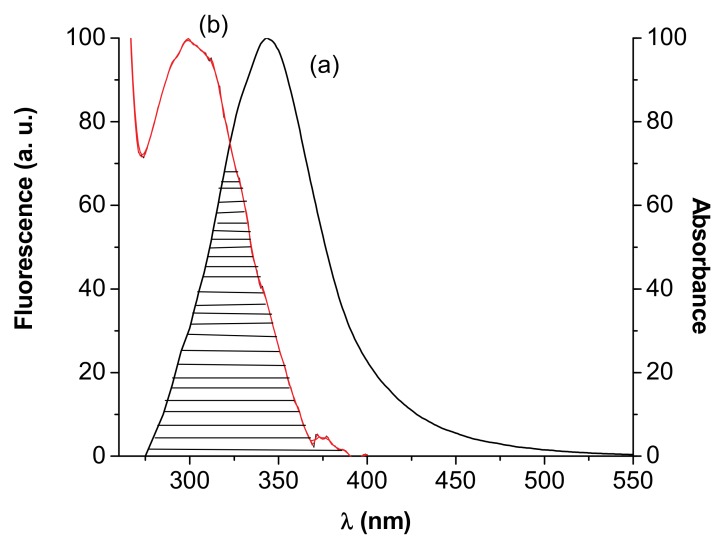
The overlap of the fluorescence spectrum of HSA (a) and the absorption spectrum of **I** (b).

The studies on fluorescence proved that BSA/ HSA could form a complex with 3-carboxy-phenoxathiin. FRET occurs whenever the emission spectrum of the fluorophore (donor) overlaps with the absorption spectrum of the acceptor. The overlap of the UV absorption spectrum of 3-carboxyphenoxathiin with the fluorescence emission spectrum of HSA is shown in Figure 8. The distance between the donor and acceptor can be calculated according to Förster’s theory [39]. The efficiency of energy transfer, *E*, is calculated using the Equation (8):

(8) $$E=1-\frac{F}{F_{0}}=\frac{R_{0}^{6}}{R_{0}^{6}+r^{6}}$$

where *F_0_* and *F* are the fluorescence intensities of BSA / HSA measured in the absence and in the presence of the ligand; *r* represents the acceptor - donor distance and *R*_0_ is the critical distance when the transfer efficiency is 50%.

(9) $$R_{0}^{6}=8.8\times 10^{-25}k^{2}n^{-4}\Phi J\;(\mathrm{in}\;cm^{6})$$

where *k*^2^ is the spatial orientation factor, which describes the relative position of the donor and acceptor dipoles, ranging from 0 (perpendicular dipoles) to 4 (parallel dipoles). Generally, the dipoles are assumed to be rapidly moving, on timescales similar to the donor excited-state lifetime, and their orientations are therefore described as random, with *k*^2^ = 2/3 [28]; *n* is the refractive index of the medium, *Φ* the fluorescence quantum yield of the donor and *J* is the overlap integral of the fluorescence emission spectrum of the donor and the absorption spectrum of the acceptor. *J* is given by:

(10) $$J=\frac{\Sigma F(\lambda )\varepsilon (\lambda )\lambda^{4}\Delta \lambda }{\Sigma F(\lambda )\Delta \lambda }$$

where *F*(*λ*) is the fluorescence intensity of the fluorescent donor at wavelength *λ* and *ε*(*λ*) is the molar absorption coefficient of the acceptor at wavelength *λ*. In the present case, *k*^2^ = 2/3, *n* = 1.36 and *Φ* = 0.15 [40].

The experimental value for the efficiency to be used in Equation (8) was estimated by measuring the fluorescence at equal protein–ligand concentration as described in [41,42,43,44]. From Equations (8)—(10), *J*, *R*_0_ (nm), *E* and *r* (nm) were calculated (Table 3).

HSA has a single tryptophan residue, Trp-214, mainly responsible for the protein’s fluorescence. In BSA, the tryptophan residues involved in binding could be either Trp-134 or Trp-212. Trp-134 is located on the surface of the albumin molecule, more exposed to a hydrophilic environment, whereas Trp-212 is deeply buried in a hydrophobic binding pocket of the protein [45,46]. So, the FRET mechanism allowed for the determination of the distance between the Trp-214/Trp-212 and the bound 3-carboxyphenoxathiin. The value obtained for the distance from the ligand to the tryptophan residue of the protein, r < 7 nm, 0.5R_0_ < r < 1.5R_0_, [47,48,49] indicated that the energy transfer from Trp-214/Trp-212 to 3-carboxyphenoxathiin occurs with high possibility.

### 2.6. Circular Dichroism Spectra

**Figure 9:**
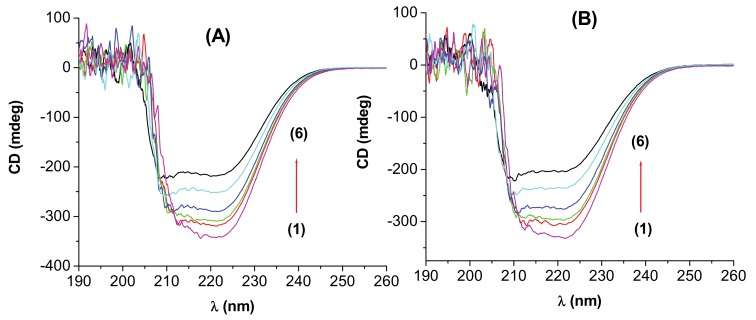
CD spectra of the (A) HSA–**I** system; (B) BSA-**I** system at pH 7.4; [HSA] =[BSA]= 3×10^-6^ M; 1–6: d/p = 0, 0.46, 0.66, 1.16, 2.33, 3.66, respectively.

The circular dichroism (CD) spectroscopy can also be used to determine the binding parameters [50] as the measured protein ellipticity depends on the ligand concentration, but the errors are usually larger than by the fluorescence method. However, the CD method is useful to ascertain the possible influence of the interaction process on the secondary structure of the proteins. Both HSA and BSA present in the ultraviolet region at 208 and 222 nm two negative bands, characteristic for the α-helical structure [42,48,51,52]. CD measurements performed in the presence of different concentrations of **I** showed that the binding of 3-carboxyphenoxathiin to BSA/HSA caused a decrease in both bands (Figure 9). The CD results were expressed in terms of mean residue ellipticity (MRE) in deg cm^2^dmol^−1^, according to the following equation:

(11) $$MRE=\frac{ObservedCD(m\deg)}{C_{p}nl\times 10}$$

where C_p_ is the molar concentration of the protein, *n* is the number of amino acid residues of the protein and *l* is the path length.

The α-helix contents of free and combined BSA / HSA were calculated from mean residue ellipticity values at 222 nm using the following equation [42]:

(12) $$\alpha -helix\%=\frac{MRE_{222}-2340}{30300}\times 100$$

At the molar ratio of drug/protein d/p = 2.33, the decrease of α-helical content is about 2.7% for BSA and about 0.81% for HSA and compound **I**.

The decrease of the CD signal indicated that the binding of **I** to the two proteins induced some conformational changes, but the secondary structure of BSA / HSA remains predominantly α-helix.

## 3. Experimental

### 3.1. Materials

Bovine serum albumin (BSA, Fraction V, approximately 99%) and human serum albumin (HSA, fatty acid free <0.05%) were purchased from Sigma Chemical Company (St Louis, MO, USA). The phenoxathiin derivative was synthesized as previously described [5]. The solutions of 3-carboxy-phenoxathiin (**I**), BSA and HSA were prepared in pH 7.4 phosphate buffer.

### 3.2. Apparatus and Methods

Fluorescence measurements were performed on a Jasco FP-6300 spectrofluorimeter. A 1.00 cm quartz cell was used for these studies. Fluorescence spectra were recorded at room temperature (25 °C) in the range 300–550 nm, upon excitation at 286 nm for each albumin. CD measurements were made on a Jasco J-815 CD spectrometer using a 1.00 cm cell at 0.2 nm intervals, with three scans averaged for each CD spectrum in the range 200–260 nm, and the results are expressed as ellipticity ([*θ*]) in millidegrees. On the basis of preliminary experiments, BSA and HSA concentrations were kept fixed at 3×10^-6^ M and the drug concentrations were varied to ensure a d/p ratio in the range 0–5.

## 4. Conclusions

The steady state fluorescence method attests a strong binding between 3-carboxyphenoxathiin in its dissociated form, the carboxylate ion and the serum albumins. It was found a one to one interaction, implying the Trp 214/212 residues and association constants in the range of 6.5×10^5^-1.1×10^6^ M^-1^. The red shifts of the maximum emission wavelengths using the synchronous fluorescence at Δλ = 60 nm reflect the change of the local polarity around the mentioned Trp residues. The circular dichroism spectra revealed changes in the protein bands upon interaction but the α-helix percent was only slightly modified. The obtained data show that this phenoxathiin derivative can be used as fluorescence probe for proteins being especially suitable for detecting the changes in the local polarity.

## Figures and Tables

**Table 1:** Quenching parameters K_SV_ and V obtained by fitting all the experimental points to eq. (2) and considering two linear segment plots for fitting to eq. (1); r^2^ represents the correlation coefficients of the plots.

	Equation (1), d/p < 1		Equation (1), d/p > 1		Equation (2)		
**Protein**	K_SV_×10^5^ (M^-1^) domain 1	r^2^	K_SV_×10^5^ (M^-1^) domain 2	r^2^	KK_SV_×10^5^ (M^-1^)	V×10^5^ (M^-1^)	r^2^
**HSA**	3.30 ± 0.03	0.999	5.60 ± 0.10	0.999	2.10 ± 0.05	0.76 ± 0.02	0.999
**BSA**	3.10 ± 0.74	0.991	7.40 ± 0.32	0.991	0.40 ± 0.10	1.90 ± 0.09	0.999

**Table 2:** Averaged binding parameters, n—the number of sites and K (M^-1^)—the association constant, obtained by linear and non linear fittings of the fluorescence data for the albumin–**I** systems.

Protein	Equation (6)		Equation (7)	
	n	K×10^5^ (M^-1^)	n	K×10^5^ (M^-1^)
HSA	0.80 ± 0.12	8.10 ± 0.02	0.70 ± 0.04	5.60 ± 0.20
BSA	1.00 ± 0.02	10.00 ± 0.30	1.30 ± 0.03	9.20 ± 0.36

**Table 3:** Energy transfer parameters: J, the overlap integral; E, the efficiency of energy transfer; *R*_0_, the critical distance; r is the distance from the ligand to the tryptophan residue of the protein.

Ligand	Protein	J (cm^3^ l mol^-1^)	E	R_0_ (nm)	r (nm)
(**I**)	BSA	1.634×10^-15^	0.500	1.862	1.862
	HSA	1.748×10^-15^	0.516	1.883	1.863
